# Trehalose enhances mitochondria deficits in human NPC1 mutant fibroblasts but disrupts mouse Purkinje cell dendritic growth *ex vivo*

**DOI:** 10.1371/journal.pone.0294312

**Published:** 2023-11-30

**Authors:** Collin M. MacLeod, Fawad A. K. Yousufzai, Liam T. Spencer, Sarah Kim, Lucianne A. Rivera-Rosario, Zerian D. Barrera, Lindsay Walsh, Claude Krummenacher, Benjamin Carone, Ileana Soto

**Affiliations:** 1 Department of Biology, Providence College, Providence, RI, United States of America; 2 Department of Biological & Biomedical Sciences, Rowan University, Glassboro, NJ, United States of America; Nathan S Kline Institute, UNITED STATES

## Abstract

Lysosomes play important roles in catabolism, nutrient sensing, metabolic signaling, and homeostasis. NPC1 deficiency disrupts lysosomal function by inducing cholesterol accumulation that leads to early neurodegeneration in Niemann-Pick type C (NPC) disease. Mitochondria pathology and deficits in NPC1 deficient cells are associated with impaired lysosomal proteolysis and metabolic signaling. It is thought that activation of the transcription factor TFEB, an inducer of lysosome biogenesis, restores lysosomal-autophagy activity in lysosomal storage disorders. Here, we investigated the effect of trehalose, a TFEB activator, in the mitochondria pathology of NPC1 mutant fibroblasts *in vitro* and in mouse developmental Purkinje cells *ex vivo*. We found that in NPC1 mutant fibroblasts, serum starvation or/and trehalose treatment, both activators of TFEB, reversed mitochondria fragmentation to a more tubular mitochondrion. Trehalose treatment also decreased the accumulation of Filipin^+^ cholesterol in NPC1 mutant fibroblasts. However, trehalose treatment in cerebellar organotypic slices (COSCs) from wild-type and *Npc1*^*nmf164*^ mice caused mitochondria fragmentation and lack of dendritic growth and degeneration in developmental Purkinje cells. Our data suggest, that although trehalose successfully restores mitochondria length and decreases cholesterol accumulation in NPC1 mutant fibroblasts, in COSCs, Purkinje cells mitochondria and dendritic growth are negatively affected possibly through the overactivation of the TFEB-lysosomal-autophagy pathway.

## Introduction

NPC (Niemann-Pick type C disease) is a lysosomal storage disease where mutations in the NPC1 gene cause the deficiency of the protein, which leads to childhood dementia and death at early/juvenile ages [1]. Early neuronal degeneration is a disease hallmark in human patients and animal models of NPC [1–3]. In NPC1 deficient mouse models, early post developmental demise of Purkinje cells (PCs) and ataxia are the main neuropathological and symptomatic features of the NPC disease [2, 4–7]. Our laboratory and others have found that NPC1 deficiency significantly affects PCs postnatal development by impairing lysosomal metabolic signaling, dendritic growth, synaptic development, and microglia function [8–11]. Deficiency of NPC1 in cells causes the overactivation of the metabolic regulator mTORC1 [11–13], which leads to the fragmentation and dysfunction of mitochondria in *in vitro* cells [13] and the transient overgrowth of PC dendrites in mice during the early stages of postnatal development [11]. This overgrowth in NPC1 deficient PCs is followed by the regression of their dendrites that is concomitant with the increased activation of the transcription factor TFEB [11]. TFEB regulates the transcription of genes associated with lysosomal biogenesis and function, and autophagy [14, 15]. The aberrant overlapping activation of mTORC1 and TFEB in NPC1 deficient PCs suggests a significant disruption of metabolic balance in these cells.

Disturbance of degradative pathways like autophagy has been linked to lysosomal storage diseases and neurodegenerative diseases [16, 17]. Basal autophagy plays significant roles in neurons, including clearance of cytotoxic materials, mitochondria turnover, and providing metabolic balance [18, 19]. In NPC, accumulation of cholesterol impairs lysosomal proteolysis leading to the buildup of autophagosomes and the blocking of the autophagy flux [17, 20]. *In vitro* experiments have shown that drug activation of the autophagy flux in NPC1 mutant cells reduces the buildup of autophagosomes and protects cells against drug induced cytotoxicity [17]. Inhibitors of mTORC1, a TFEB inhibitor, can prevent the accumulation of damaged mitochondria and restore lysosomal proteolysis in NPCs by facilitating the production of healthy lysosomes and inhibiting protein translation [13]. However, the biogenesis of new lysosomes allowed by these inhibitors does not fix or eliminate the accumulation of cholesterol [13, 17]. On the other hand, the hydroxypropyl-beta cyclodextrin oligosaccharide blocks autophagy flux, but significantly reduces the accumulation of lysosomal cholesterol leading to the amelioration of cellular pathology associated with the NPC disease [17].

Here we used *in vitro* and *ex vivo* models, to ask if mitochondria pathology caused by NPC1 deficiency could be corrected by activators of TFEB. Using human NPC1 mutant fibroblasts, we tested serum starvation and trehalose as inducers of TFEB activation. Trehalose is a non-reducing disaccharide of glucose, α-D-glucopyranosyl α-D-glucopyranoside, that induces autophagy through the AKT-TFEB activation pathway independently from the mTORC1 pathway [21, 22]. Treatment with trehalose was also tested in cerebellar organotypic slices (COSCs) from wild type (WT) and *Npc1*^*nmf164*^ mutant mice where PCs dendrites were developing. Our results show that trehalose effectively reversed pathological changes in mitochondria associated with the NPC1 mutation in the fibroblasts, but not in the NPC1 mutant COSCs. In fact, treatment with trehalose was detrimental to the developing PCs in WT and NPC1 mutant COSCs.

## Material and methods

### Fibroblasts cell cultures

#### Cell lines and culturing conditions

Skin derived fibroblasts from a healthy patient (HT, GM01652, F, 11YR) and a patient with a NPC1 mutation suffering from NPC (NPC1, GM03123, F, 9YR), were obtained from the Coriell Institute. HT and NPC1 fibroblasts were grown in Corning® DMEM [+] 4.5 g/L glucose, L-glutamine, sodium pyruvate, supplemented with 15% fetal bovine serum (FBS), and incubated in a humidified atmosphere containing 5% CO_2_ at 37°C. Cells were passaged when confluent. Cells between the 4th and 9th passage were used for the experiments reported here; HT and NPC1 cells were at equivalent passages.

#### Serum starvation and trehalose treatment

For the serum starvation and trehalose treatment experiments, cells were seeded in 8 well cell culture chamber slides at moderate confluency in complete media (CM: Corning® DMEM [+] 4.5 g/L glucose, L-glutamine, sodium pyruvate, supplemented with 15% FBS). Then, after 16hrs, cell medium was changed to one of the following: new CM, serum starved media (SSM), SSM + 100mM trehalose (VWR), SSM + 100mM sucrose, or CM + 100mM trehalose; and incubated in the replaced media for 24hrs. For some experiments, new CM was reintroduced for 3hrs to the cells after 24hrs with the media listed above. After the 3hrs incubation, cells were briefly washed with 1X PBS, fixed in 4% Paraformaldehyde (PFA) for 10min, and washed with PBS.

#### Immunofluorescence experiments for fibroblasts cell cultures

After fixation, HT and NPC1 fibroblasts were permeabilized with 0.5% TritonX-100 for 10 min and blocked in 5% normal donkey serum/1X PBS with 0.05% Tween-20 for 1hr, all at room temperature. Then, cells were incubated overnight at 4°C with a solution containing primary antibodies diluted in 1X PBS. Primary antibodies used for these experiments are rabbit anti-pyruvate dehydrogenase-E1 alpha (PDHE1, 1:200) and mouse anti-acetylated tubulin (AcTub 1:200). After the overnight incubation, cells were washed three times for 5 min, and incubated with appropriate secondary antibodies for 30 min at room temperature. After three washes with 1X PBS, cells were incubated with DAPI, and mounted with Aqua-Poly/mount.

#### Filipin staining

Filipin staining was performed in cells fixed with 4% paraformaldehyde and rinsed with PBS. Filipin complex ready-made solution (Sigma-Aldrich) was diluted 50ug/ml and added to the fixed cells which were incubated for 1–2 hrs. Then, cells were washed with PBS and mounted in Aqua-Poly/mount.

#### Microscope imaging and analysis of cultured fibroblasts

Immunolabeled fibroblasts with PDHE1 and AcTub were imaged using a Nikon A1R Confocal System equipped with Live Cell 6 Laser Line and Resonant Dual Scanner and 1μm images were taken with a 40X objective. To determine the volume of PDHE1^+^ mitochondria per cells, the Surface tool from the Bitplane Imaris™ software (Oxford Instruments) was first used by selecting the AcTub immunostaining to create the surface and calculate the cell area. Then, the AcTub surface was used to segregate the PDHE1 immunoreactivity, create a new surface for the PDHE1, and calculate the volume of PDHE1^+^ mitochondria. To calculate the length of mitochondria in fibroblasts, the visual magnification of the PDHE1^+^ mitochondria was increased to facilitate the ImageJ manual quantification by taking high magnified snapshots of the confocal images using the Bitplane Imaris™ software. The scale bars of the snapshots were then used to manually measure the length of mitochondria using the ImageJ software. Three images were taken per culture at 40X.

Three images were taken per culture at 40X. To measure the intensity density of Filipin^+^ cholesterol, confocal images were used, and 28–30 cells were selected using ImageJ threshold selection. Along with the Filipin intensity density, the area per cell was also measured using the AcTub immunoreactivity, then the Filipin intensity density values were divided by the cell area to calculate the Filipin immunoreactivity per cell.

### Cerebellar organotypic slice cultures

#### Animals

Mouse experiments were conducted in accordance with policies and procedures described in the Guide for the Care and Use of Laboratory Animals of the National Institutes of Health and were approved by the Animal Care and Use Committees at the Rowan University School of Osteopathic Medicine and Providence College. The results and experiments of this study that involve animals are also reported in accordance with ARRIVE 2.0 guidelines. The C57BL/6J-*Npc1*^*nmf164*^/J mouse strain (Jax stock number 004817) was provided by Dr. Robert Burgess at The Jackson Laboratory. *Npc1*^*nmf164*^ heterozygous mice were bred and housed in a 12/12-hour light/dark cycle to generate both WT and *Npc1*^*nmf164*^ homozygous mutant mice. To produce NPC1 deficient mice with PCs expressing GFP (*Npc1*^*nmf164*^*-Pcp2*^*EGF*^), *Npc1*^*nmf164*^ heterozygous mice were intercrossed with the B6;FVB-Tg(Pcp2-EGFP)2Yuza/J (Jax stock number 004690).

#### Cerebellar organotypic slice culture

For the culturing of cerebellar organotypic slices, the protocol described by Uesaka et al., was followed with some exceptions [23]. Briefly, after euthanasia, postnatal mouse brains at day 10 (P10) were dissected and kept in cold HBSS. Then, the cerebellum was separated from the rest of the brain and the portion of the brain stem along with the meninges were carefully removed while keeping the tissue immersed in HBSS. Using a tissue slicer (Stoelting), 250μm slices were cut and plated on a membrane filter (Millicell-hydrophilic PTFE 0.4um; Millipore) coated with rat-tail collagen and immersed in slice medium (50% MEM, 25% horse serum (Invitrogen), 25% HBSS, 3mM GlutaMAX (Invitrogen), and 5 mg/ml glucose). However, Mifepristone, which is usually used to help the survival of Purkinje cells [23], was not used in the experiments reported here. The slices were cultured for 4 days at 37°C in an environment of humidified 95% air and 5% CO2. The medium was changed every other day, and slices were washed and fixed with 4% PFA for 30 min on day 4 *in vitro*. For Trehalose treatment, the disaccharide was dissolved in the slice medium at 100nM concentration.

#### COSCs immunohistochemistry

Using a scalpel, the membrane with the slices still attached were cut and, using a brush, transferred into the wells of a 24-well plate containing 1x PBS. The PBS was then replaced with pre-cooled 100% ethanol and slices were incubated at -20°C for 15 to 20 min. The slices were washed three times in 1x PBS for 10 min at room temperature and incubated for 30 min with a solution containing 1x PBS + 1% Triton-X 100 (1%PBT) and 20% NDS detergent at room temperature. Next, the slices were incubated overnight with the primary antibodies diluted in blocking solution at 4°C. The primary antibodies used on cerebellar organotypic slices were rabbit anti-pyruvate dehydrogenase E 1 alpha (PDHE1) (1:200, GeneTex, # GTX104015), rabbit anti-TFEB (1:200, Bethyl Laboratories, A303-673A), LAMP1 (1:200, BioLegend, 121602) and mouse anti-CALB (calbindin,1:200, Sigma-Aldrich) when PCP2-GFP expression was not used. The corresponding secondary antibodies were then used after three washes with 1X PBT (1:500, Jackson-ImmunoResearch or Invitrogen). Cerebellar sections were then washed three times with 1X PBT for 10–15 min, incubated with DAPI, and mounted in Aqua-Poly/mount (Polysciences).

#### Microscope imaging and analysis of COSCs

To quantify PDHE1^+^ mitochondria length, similar analysis as described above for fibroblasts was performed for COSCs. Briefly, immunolabeled COSCs with PDHE1 and CALB were imaged using a Nikon A1R Confocal System equipped with Live Cell 6 Laser Line and Resonant Dual Scanner and 1μm images were taken with a 40X objective. To isolate the PDHE1^+^ mitochondria from CALB^+^ PCs, the Surface tool from the Bitplane Imaris™ software (Oxford Instruments) was first used by selecting the CALB immunostaining to create the surface of the PCs. Then, the CALB surface was used to segregate the PDHE1 immunoreactivity (by masking it and creating a new channel). To calculate the length of mitochondria in PCs, the visual magnification of the channel containing the PC specific PDHE1^+^ mitochondria was increased to facilitate the ImageJ manual quantification, then high magnified snapshots of the confocal images were taken using the Bitplane Imaris™ software. The scale bars of the snapshots were then used to manually measure the length of mitochondria using the ImageJ software. Three images were taken per culture at 40X.

Quantifications of the percentage of the LAMP1 total volume inside CALB^+^ PCs soma and dendrites were performed as previously reported [11]. Using the Imaris software and 40X confocal images, a 3D surface rendering was created for the CALB channel, then the total volume (sum) of CALB^+^ dendrites was measured. Then, the LAMP1^+^ structures inside of the created CALB surface were masked to create a new channel after clearing all the fluorescence that is not found overlapping/contacting the CALB rendering surface. Then, 3D surface renderings were created for the newly created LAMP1 channels in order to determine the sum volume of these immunostained structures. The Imaris™ software calculated and provided the measurements of the respective volumes for CALB and LAMP1, and the percentages of these markers in PC dendrites were calculated by dividing them by the CALB volume of the dendrites.

To determine changes in dendritic tree morphology, COSCs were imaged using a confocal microscope. A stack of 4 confocal image slices (each 1 um in depth) were combined to make 1 3D image per COSC. Each 3D image was cropped to 147um by 98.7 um by 4 um (S1 Fig). Quantitative analysis of 3D dendritic morphology was performed using the Filaments tracer tool of the Imaris 9.9.1 software (S1 Fig). Erroneous filament segments were deleted using the Filaments Edit function. Each analysis used 9–10 NPC COSC, 6 TH COSC, and 7–8 WT COSC confocal images.

To calculate the percentage of CALB^+^ PCs in COSCs with nuclear immunoreactivity of TFEB, 8 confocal images were taken per genotype (WT and *Npc1*^*nmf164*^). The total number of CALB^+^ PCs and the number of CALB^+^ PCs with nuclear TFEB immunoreactivity was manually counted using the Bitplane Imaris software.

### Statistical analysis

Data were analyzed using GraphPad Prism software. Significance was calculated using unpaired t-tests for comparisons between two groups. p-values are provided as stated by GraphPad Prism software and significance was determined with p-values less than 0.05.

## Results

### NPC1 mutant fibroblasts with mitochondrial deficits have a proper response to serum starvation

**Fig 1:**
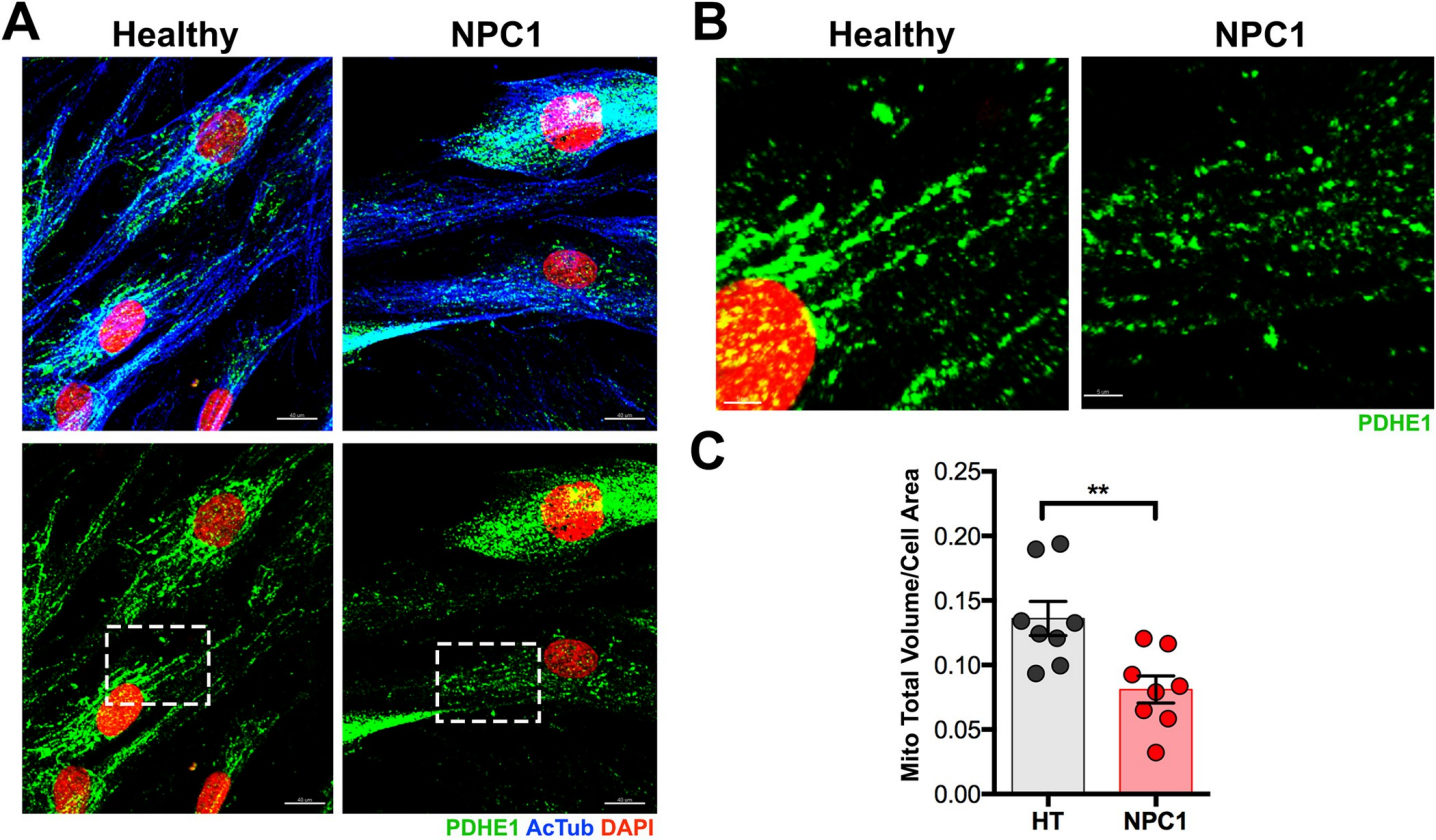
The total volume of PDHE1^+^ mitochondria is reduced in NPC1 mutant fibroblasts. A. Healthy and NPC1 mutant fibroblasts immunolabeled with PDHE1, AcTub and DAPI. B. Inserts from (A) showing the PDHE1 immunoreactivity in Healthy and NPC1 mutant fibroblasts at a higher magnification. C. Quantitative analysis showing a significant decrease in the total volume of PDHE1^+^ mitochondria per cell area in NPC1 mutant fibroblasts when compared to Healthy (HT) cells. Data are presented as mean ± SEM, n = 8 cells. **P < 0.01. Scale bar: (A) 50 μm (B) 10 μm.

**Fig 2:**
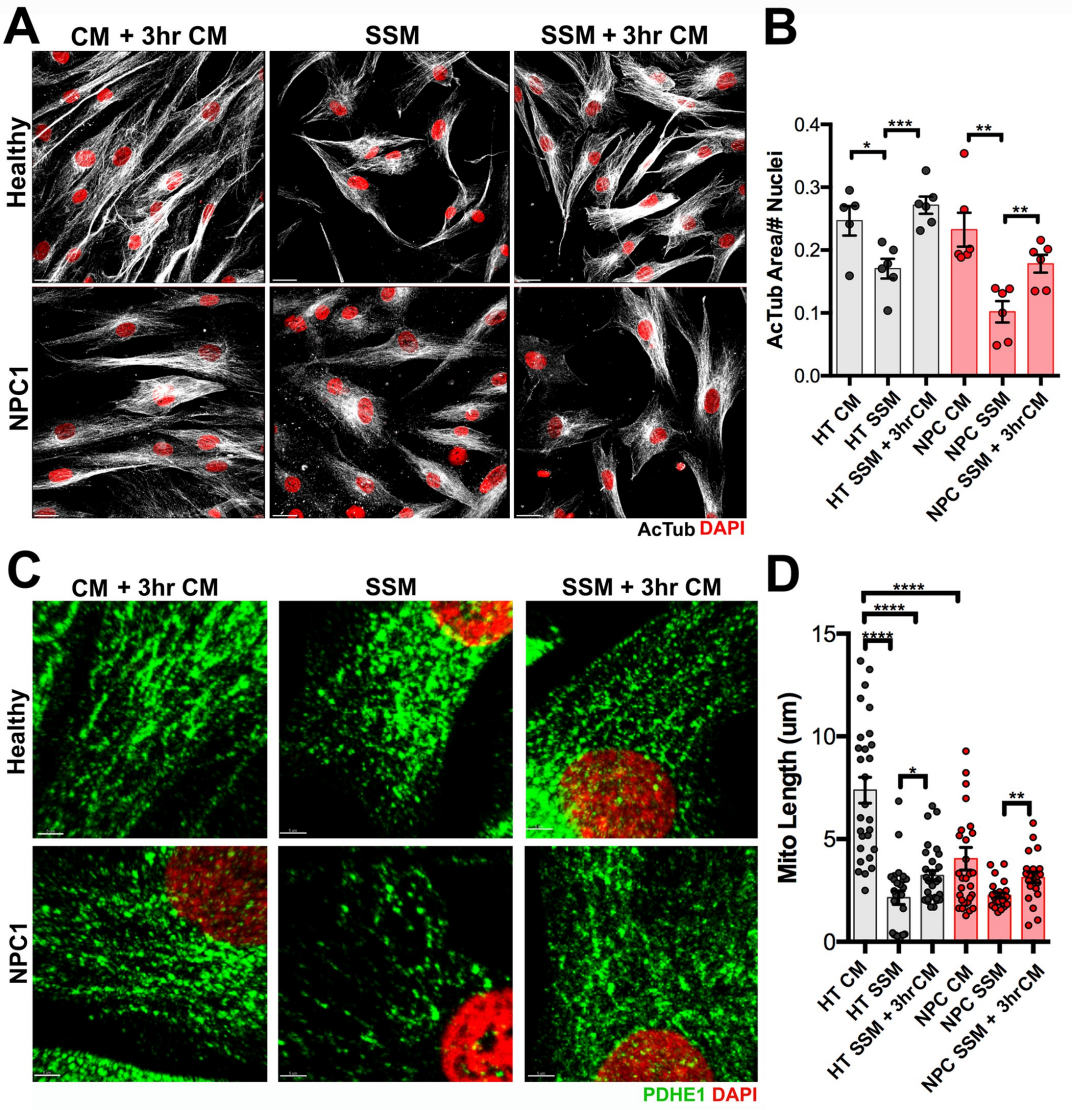
NPC1 mutant fibroblasts and their fragmented mitochondria have a proper response to serum withdrawal and reintroduction. A. Healthy and NPC1 mutant fibroblasts immunolabeled with AcTub and DAPI showing morphological changes after 24hrs in complete media (CM) + 3hr CM, serum starved media (SSM), and SSM + 3hr CM. B. Quantitative analysis of the total cellular area per cell (AcTub area/#nuclei) after the different treatments described on (A). C. Healthy and NPC1 mutant fibroblasts immunolabeled with PDHE1 and DAPI showing mitochondrial morphological changes after 24hrs of CM + 3hr CM, SSM, and SSM + 3hr CM. D. Quantitative analysis of the length of PDHE1^+^ mitochondria in HT and NPC1 mutant fibroblasts after the different treatments described above. Data are presented as mean ± SEM, (B) n = 6 images, (D) n = 28–30 mitochondria per genotype/treatment. *P < 0.05, **P < 0.01, ***P < 0.001, ****P < 0.0001. Scale bar: (A) 30 μm (C) 5 μm.

*In vitro* and *in vivo* studies have demonstrated that NPC1 deficient cells present significant mitochondrial deficits and dysfunction [11, 13, 24–26]. For instance, NPC1 mutant fibroblasts not only have alterations in mitochondria organization and composition of the respiratory chain complex, but also a significant reduction in cellular ATP with an increased respiration rate [24]. By using antibodies against the Pyruvate dehydrogenase e1 alpha (PDHE1) to label mitochondria and acetylated Tubulin (AcTub) to label cell area, we quantified the total volume of mitochondria per cell area in healthy (HT) and NPC1 mutant human fibroblasts (Fig 1A). As expected, the total volume of all mitochondria that occupy the area of each cell was significantly lower in NPC1 mutant fibroblasts than in HT fibroblasts (Fig 1A–1C). It was also noticed that PDHE1^+^ mitochondria were more globular and fragmented in NPC1 mutant fibroblasts when compared to HT fibroblasts as previously reported by others [13, 24, 26]. Given that nutritional and metabolic changes affect cellular and mitochondrial morphology [27, 28], we interrogated if NPC1 mutant fibroblasts and their mitochondria would have a similar response to serum starvation as HT fibroblasts. For instance, serum starvation causes cellular shrinkage [27] and mitochondria fragmentation [28, 29]. After 24hrs in serum starved media (SSM), we found that both HT and NPC1 mutant fibroblasts had a significant reduction of Ac-Tub^+^ cellular area when compared with fibroblasts in complete media (CM) (Fig 2A and 2B). Similarly, when CM was introduced for 3hrs after 24hrs of serum starvation (SSM + CM), HT and NPC1 mutant fibroblasts were able to increase their Ac-Tub^+^ cellular area to levels that were statistically higher than SSM levels but not different from levels in CM (Fig 2A and 2B). These results showed that NPC1 mutant fibroblasts can properly respond to serum starvation and reintroduction as HT fibroblasts do.

Next, we investigated if mitochondrial response to serum starvation was altered in NPC1 mutant fibroblasts. This time, the length of PDHE1^+^ mitochondria was measured using 3D confocal images, and the Imaris and ImageJ software. As previously reported, we found that mitochondria from HT fibroblasts significantly reduced their length from a long tubular shape to a more globular fragmented morphology after 24hrs of serum starvation (Fig 2C and 2D). However, when serum was re-introduced for 3hrs to HT fibroblasts after 24hrs starvation, a small but significant increase in mitochondria length was found when compared to HT fibroblasts in SSM, but still significantly lower when compared to mitochondria in non-starved (CM) HT fibroblasts (Fig 2C and 2D), indicating that a longer time of CM may be needed for complete recovery of tubular elongated mitochondria. The mitochondria length of NPC1 fibroblasts was significantly shorter than the HT fibroblasts mitochondria length in CM (Fig 2C and 2D). After 24hrs of serum starvation, in NPC1 fibroblasts the reduction of mitochondria length was not significant when compared to CM NPC1 fibroblast mainly because of the variability in mitochondria fragmentation in the CM group. However, when CM was re-introduced to starved NPC1 mutant fibroblast, a significant increase in mitochondrial length was quantified in this group (SSM + 3hrs CM) when compared to the serum starved (SSM) NPC1 mutant fibroblasts (Fig 2C and 2D). Overall, these results showed that NPC1 mutant fibroblasts are able to change and adapt to serum starvation and reintroduction similarly as HT fibroblasts by reducing or increasing cell size and mitochondria length.

### Trehalose treatment induces tubular and elongated mitochondria in NPC1 mutant fibroblasts

**Fig 3:**
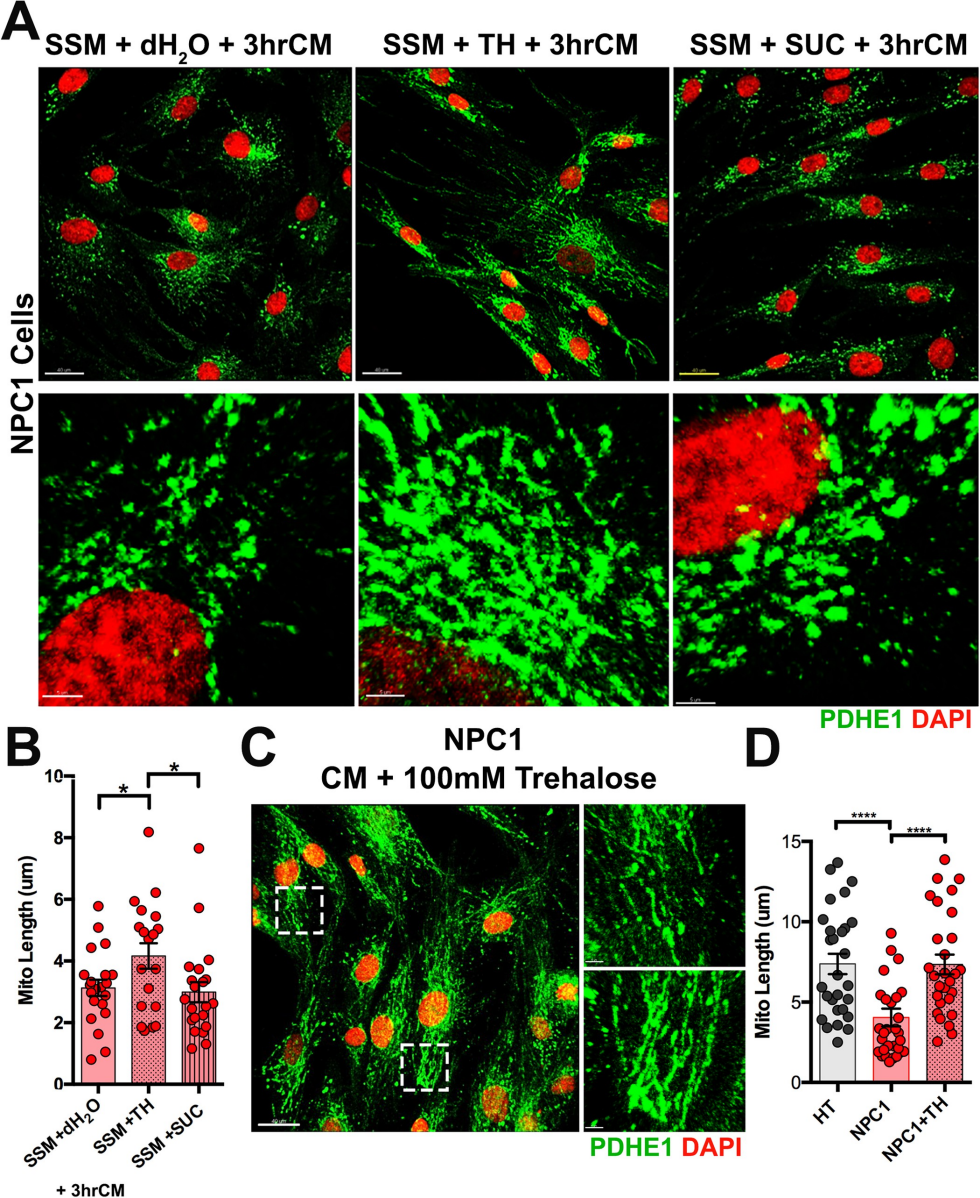
Trehalose treatment induces tubular and elongated mitochondria in NPC1 mutant fibroblasts. A. NPC1 mutant fibroblasts immunolabeled with PDHE1 and DAPI showing morphological changes in mitochondria after 24hrs of SSM supplemented with H_2_O as a vehicle, 100mM Trehalose, or 100mM Sucrose plus 3hrs of CM. B. Quantitative analysis of the length of PDHE1^+^ mitochondria in NPC1 mutant fibroblasts after the different treatments described in (A). C. NPC1 mutant fibroblasts immunolabeled with PDHE1 and DAPI showing mitochondrial morphological changes after 24hrs of CM + 100mM TH (inlets). D. Quantitative analysis of the length of PDHE1^+^ mitochondria from HT and NPC1 mutant fibroblasts showing that CM supplemented with TH induces mitochondria lengths in NPC1 fibroblasts similar to HT cells. *Data are presented as mean ± SEM, (B) n = 20–21 mitochondria/treatment, (D) n = 28 mitochondria/genotype-treatment. *P < 0.05, ****P < 0.0001. Scale bar: (A) 40 μm and 5 μm (C) 40 μm and 5 μm.

It has been suggested that an interplay between mitophagy and mitochondrial biogenesis is necessary to prevent pathological conditions associated with mitochondria dysfunction [30, 31]. Accumulation of fragmented and damaged mitochondria has been found in Purkinje cells from NPC1 mutant mice and *in vitro* NPC1 deficient cellular models where lysosomal degradation is severely impaired [11, 13, 24]. In this study, NPC1 mutant fibroblasts were able to increase mitochondrial length after serum starvation and reintroduction of CM, suggesting that a potential increase in lysosomal activity induced by serum starvation enhanced the fusion or biogenesis of mitochondria when CM was reintroduced. In fact, we found nuclear TFEB immunoreactivity in serum starved HT and NPC1 fibroblasts (S2 Fig), which could indicate that lysosomal activity was enhanced by starvation. Therefore, we decided to use trehalose to further increase the lysosomal activity. Trehalose is a well-known activator of TFEB [22, 32], which has improved mitochondria morphology in human fibroblasts with an ataxia-associated mutation in the C-terminal HSP70-interacting protein (CHIP) [33]. NPC1 mutant fibroblasts were treated for 24hrs with 100mM trehalose (TH) while in SSM, then CM was reintroduced for 3hrs, and mitochondria length was measured. We found that TH treatment significantly increased mitochondrial length in NPC1 mutant fibroblasts when compared to the same type of cells treated with water (SSM + H2O) or 100mM sucrose (SSM + SUC), when CM was reintroduced for 3hrs (Fig 3A and 3B). Furthermore, addition of TH to NPC1 mutant fibroblasts while in CM for 24hrs drastically increased mitochondria length comparable to HT fibroblasts under normal conditions (CM) (Fig 3C and 3D). Addition of TH to SSM did not alter mitochondria length in HT cells after CM was reintroduced for 3hrs when compared to untreated HT fibroblasts (S3 Fig).

**Fig 4:**
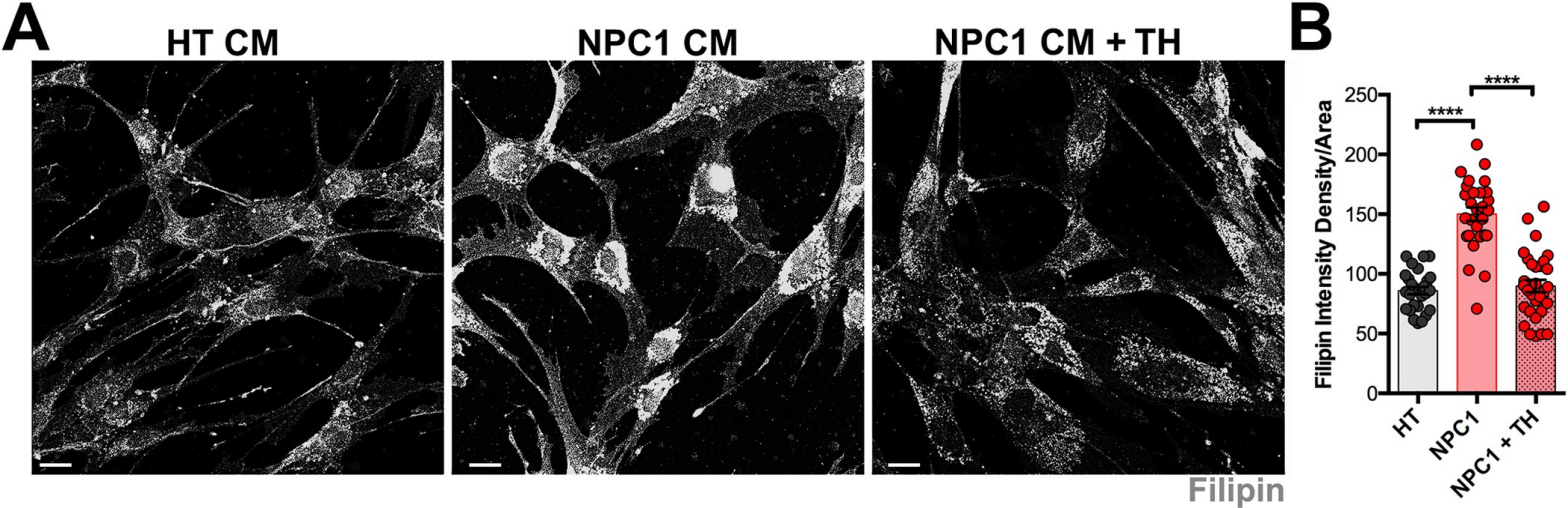
Cholesterol accumulation is reduced in NPC1 mutant fibroblasts after trehalose treatment. A. HT and NPC1 mutant fibroblasts in CM or CM + 100mM TH stained with Filipin. B. Quantitative analysis of the Filipin intensity density per area. Data are presented as mean ± SEM, n = 28–30 cells. ****P < 0.0001. Scale bar: (A) 30 μm.

It is thought that TH promotes TFEB activation by directly impacting lysosomes, specifically by causing lysosomal enlargement and membrane permeabilization [32]. TFEB activation also improves lysosomal function [34, 35]. Both, HT and NPC1 mutant fibroblasts treated with TH showed TFEB nuclear immunoreactivity (S4 Fig). Given that NPC1 mutation causes impaired lysosomal degradation and the accumulation of cholesterol, we interrogated if TH treatment effects on mitochondria health of NPC1 mutant fibroblasts were due to changes in lysosomal accumulation of cholesterol. In fact, we found that the high levels of Filipin^+^ cholesterol in NPC1 mutant fibroblasts were significantly reduced to levels found in HT fibroblasts when treated with 100mM TH (Fig 4A and 4B). These results suggest that TH treatment induces tubular and elongated mitochondria in NPC1 mutant fibroblasts by enhancing the clearance of the pathological lysosomal accumulation of cholesterol in these cells.

### NPC1 deficient Purkinje cells have shorter and fragmented mitochondria *ex-vivo*

**Fig 5:**
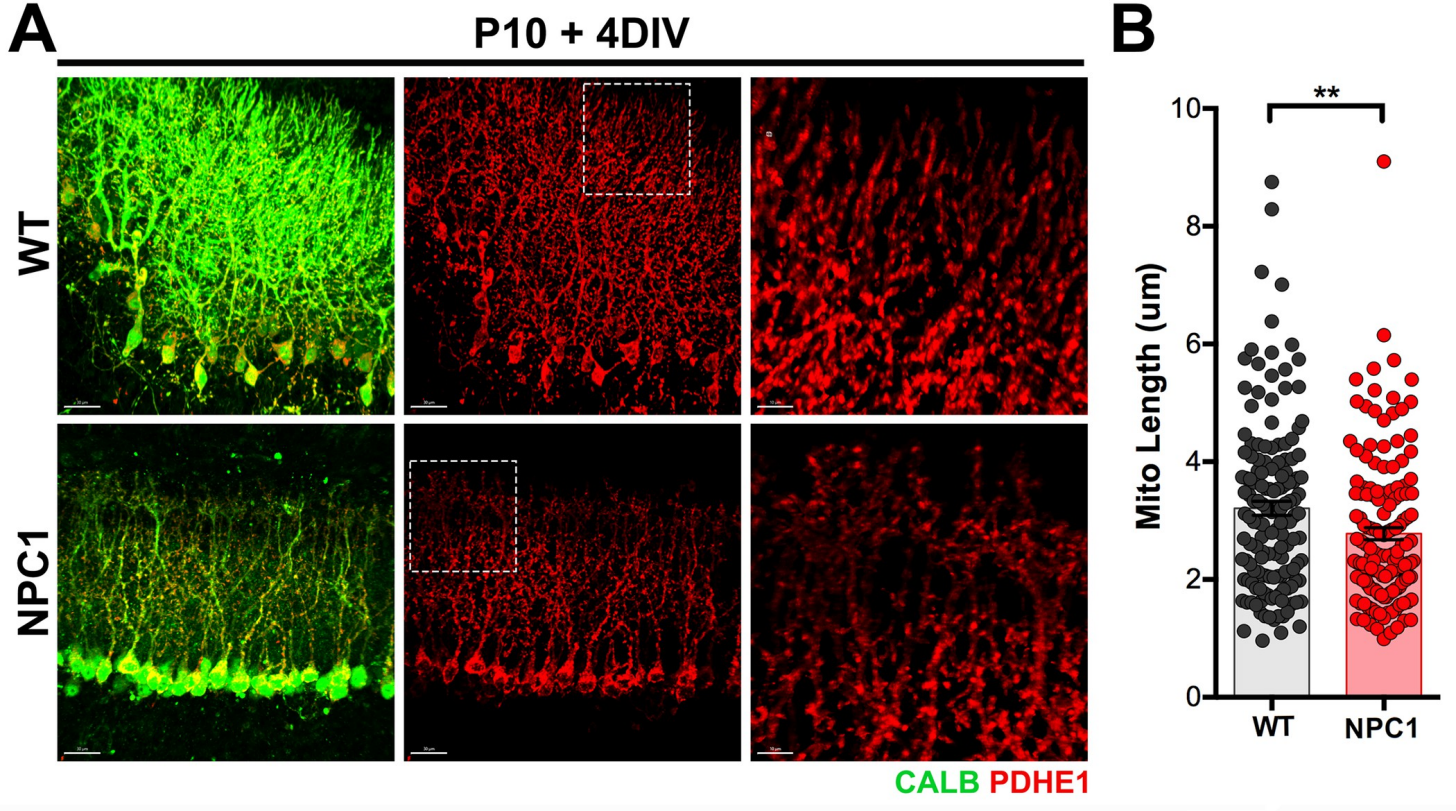
Fragmented PDHE1^+^ mitochondria in *Npc1*^*nmf164*^ Purkinje cells *ex vivo*. A. WT and *Npc1*^*nmf164*^ cerebellar organotypic slices cultured for 4 DIV immunolabeled with PDHE1 and CALB. B. Quantitative analysis of the length of PDHE1^+^ mitochondria in WT and *Npc1*^*nmf164*^ Purkinje cells from COSCs after 4 DIV. Data are presented as mean ± SEM, n = 150 mitochondria from n = 3 COSC s/genotype. **P < 0.01. Scale bar: (A) 30 μm and 10 μm (third column).

NPC1 deficiency leads to the early degeneration of neurons in Niemann-Pick Type C disease. Given that reduced levels and fragmentation of mitochondria are found in Purkinje cells (PCs) from *Npc1*^*nmf164*^ mutant mice [11], we pursued to determine the effects of TH treatment in NPC1 mutant PCs. Cerebellar slices at postnatal day 10 (P10) from wild type (WT) and *Npc1*^*nmf164*^ mutant mice were used for organotypic slice cultures, and after 4 days *in vitro* (DIV) were fixed and immunolabeled with calbindin (CALB) and PDHE1 antibodies. Using the Imaris software, PDHE1^+^ mitochondria from CALB^+^ PCs were segregated, and mitochondria length was measured in WT and *Npc1*^*nmf164*^ samples (Fig 5A). We found that *Npc1*^*nmf164*^ PCs from P10 + 4DIV cerebellar organotypic slice cultures (COSC) had a significant decrease in mitochondria length when compared to WT PCs (Fig 5A and 5B).

### Trehalose treatment causes mitochondria fragmentation and impairs Purkinje cells dendritic growth *ex-vivo*

**Fig 6:**
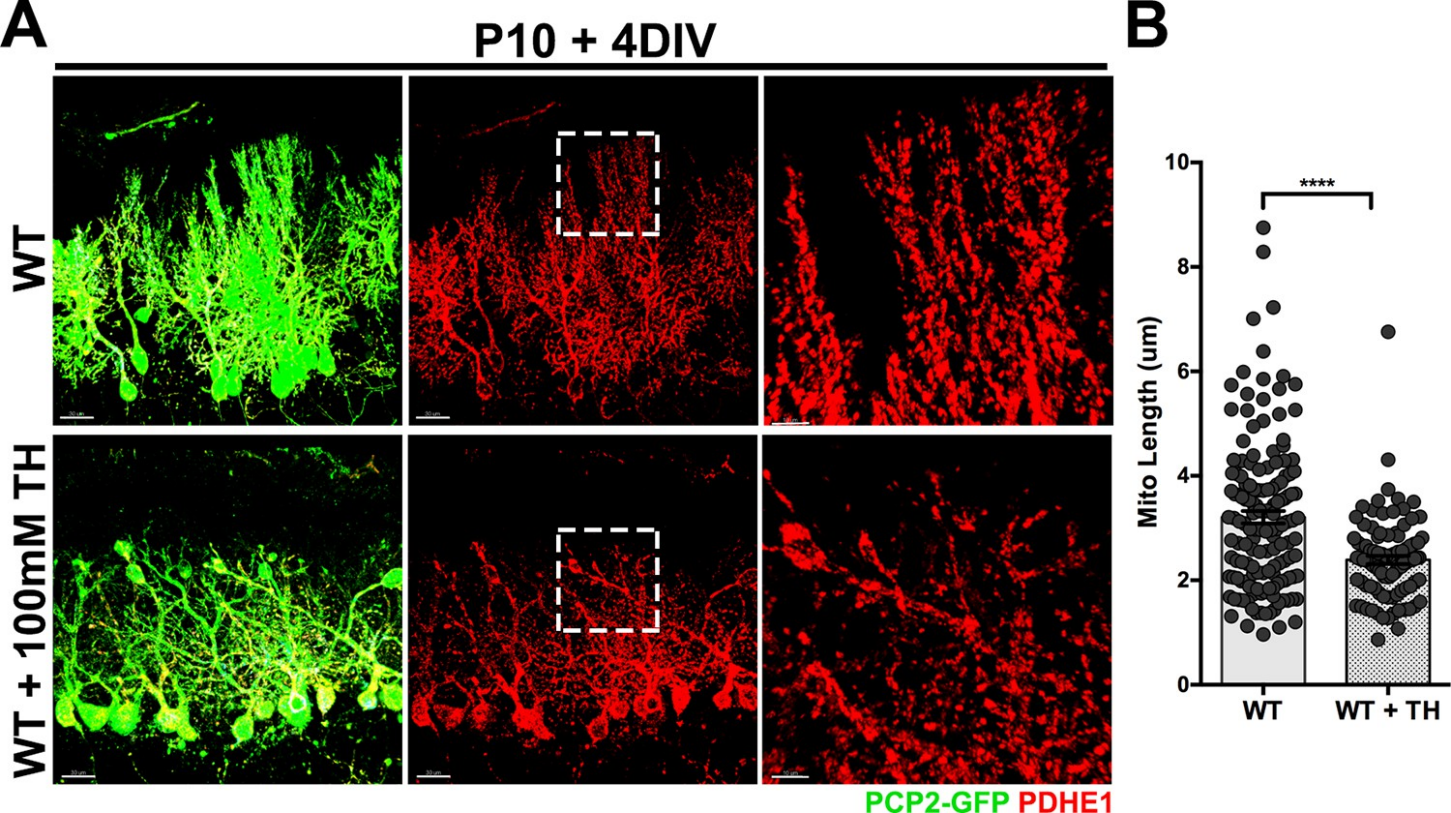
Trehalose treatment induced fragmented PDHE1^+^ mitochondria in Purkinje cells *ex vivo*. A. Non-treated and TH treated PCP-2-GFP WT Purkinje cells from P10 cerebellar organotypic slices cultured for 4 DIV immunolabeled with PDHE1. B. Quantitative analysis of the length of PDHE1^+^ mitochondria in non-treated and TH treated PCP-2-GFP WT Purkinje cells from P10 + 4DIV COSCs. Data are presented as mean ± SEM, n = 6–10 cells. ****P < 0.0001. Scale bar: (A) 30μm and 10μm (third column).

**Fig 7:**
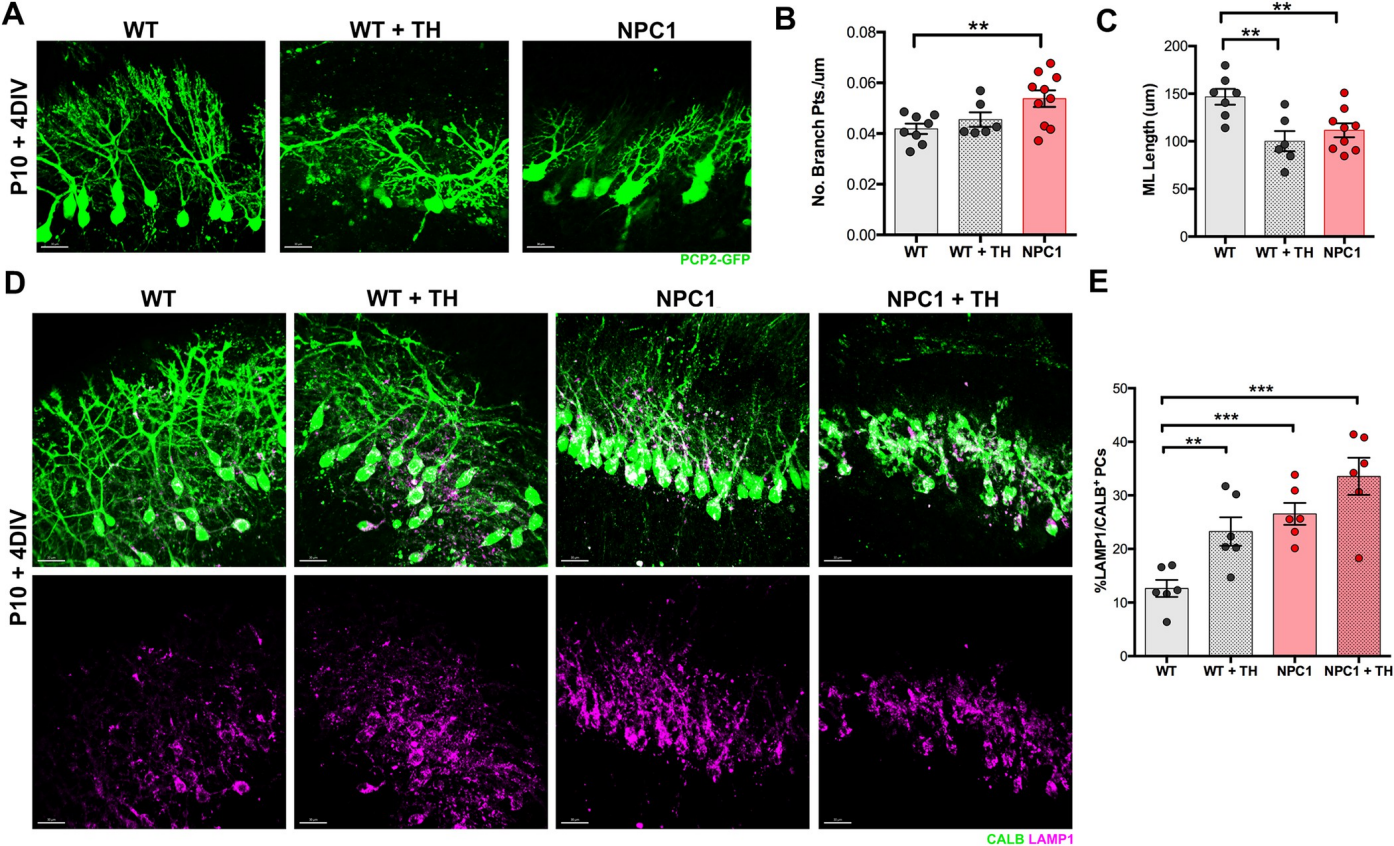
Trehalose treatment induced reduced dendritic growth and increased total volume of LAMP1^+^ lysosomes in Purkinje cells *ex vivo*. A. PCP-2-GFP^+^ Purkinje cells from WT, TH treated WT, and NPC1 P10 + 4DIV COSCs showing dendritic growth and branching. B. Quantitative analysis of the number of branch points per micrometer in PCP-2-GFP^+^ Purkinje cells from WT, TH treated WT, and NPC1 P10 + 4DIV COSCs. C. Quantitative analysis of the length of the molecular layer in PCP-2- GFP^+^ Purkinje cells from WT, TH treated WT, and NPC1 P10 + 4DIV COSCs. Data are presented as mean ± SEM, n = 6–10 cells. D. WT, WT + TH, NPC1, and NPC1 + TH P10 + 4 DIV COSCs immunolabeled with LAMP1 and CALB. LAMP1 immunoreactivity from CALB^+^ PCs was isolated using an Imaris masking tool. E. Quantitative analysis of the percentage of the LAMP1^+^ lysosomes total volume in CALB^+^ PCs from WT, WT + TH, NPC1, and NPC1 + TH COSCs after 4 DIV. Data are presented as mean ± SEM, n = 6 images, n = 3 COSCs. **P < 0.01, ***P < 0.001, ****P < 0.0001. Scale bar: (A) and (D) 30μm.

Next, we added 100mM TH to the COSC media and cultured for 4 days P10 cerebellar slices from PCP2-GFP-WT and PCP2-GFP*-Npc1*^*nmf164*^ mice. We found that the length of PDHE1^+^ mitochondria was significantly reduced in PCP2-GFP^+^ PCs from TH treated WT when compared to non-treated WT COSCs (Fig 6A and 6B). TH treatment in PCP2-GFP*-Npc1*^*nmf164*^ COSCs led to a severe degeneration of PCs (S5 Fig), so PDHE1^+^ mitochondria length was not quantified in these samples. Measurements of the ratio of dendritic tree branching points per length (μm) in PCP-2-GFP^+^ PCs from WT, WT TH treated, and NPC1 mutant COSCs showed a higher number of branch points per um in *Npc1*^*nmf164*^ PCP2-GFP^+^ PCs when compared to WT and WT TH treated COSCs (Fig 7A and 7B). No significant differences in branch points/um were found in PCP2-GFP PCs from WT and WT TH treated COSCs (Fig 7A and 7B). However, the length of the molecular layer (ML) was significantly reduced in PCP2-GFP^+^ PCs from WT TH treated, and *Npc1*^*nmf164*^ mutant COSCs when compared to PCP2-GFP^+^ PCs from non-treated WT COSC (Fig 7A and 7C). These data show that TH treatment in WT COSC affects the vertical growth of PCs dendrites (shortens ML), while NPC1 deficiency in PCs affects the vertical growth of dendrites (shortens ML) and increases the addition of branches. We also confirmed that TH treatment increased the nuclear translocation of TFEB in PCs from WT TH treated COSC (S6 Fig). Additionally, a higher percentage of the total volume of LAMP1^+^ lysosomes per the total volume of CALB^+^ PCs was found in *Npc1*^*nmf164*^ and TH treated (WT and *Npc1*^*nmf164*^) COSCs when compared to untreated WT COSCs (Fig 7D and 7E). Our data suggest that dendritic changes in *Npc1*^*nmf164*^ and TH treated COSCs are associated with increased lysosomal activity that could be driven in part by the activation of TFEB.

## Discussion

Lysosomal function has a central role in degradation of protein and cellular components, nutrient sensing, and metabolic signaling and balance [36, 37]. Lysosomes support the autophagy pathway through their cellular clearance function and the transcriptional regulation of genes associated with autophagy and lysosomal biogenesis [37]. Therefore, autophagy is significantly disrupted in lysosomal storage diseases like NPC [16]. In fact, accumulation of autophagosomes and lack of lysosomal proteolysis lead to the disruption of the autophagy pathway and function [17, 20]. Moreover, impaired mitophagy is implicated in the accumulation of damaged and fragmented mitochondria in NPC1 deficient cells [13]. Given that lysosomal dysfunction in NPC affects autophagy and mitophagy, we interrogated if NPC1 mutant fibroblasts would be able to respond appropriately to metabolic stressors, such as serum starvation. First, we confirmed that the volume of mitochondria per cell area is significantly decreased in NPC1 mutant fibroblasts as previously reported [24] and similar to NPC1 deficient neurons [11, 13]. Then, HT and NPC1 mutant fibroblasts were cultured for 24hrs in serum starved media to determine if NPC1 mutant fibroblasts would respond differently than HT fibroblasts. Serum starvation in fibroblasts induces the Warbug effect, which is the usage of the anaerobic glycolysis pathway even when oxygen is present [38]. Because of this metabolic shift, fibroblasts become quiescent by stopping proliferation and reducing their cellular area [27, 28]. We found that both HT and NPC1 mutant fibroblasts were able to reduce their cellular area during the withdrawal of serum and to recover their previous size once serum was introduced again for three hours.

Another expected consequence of serum starvation is the activation of TFEB and autophagy, which acts as a nutrient supplier through the degradation and reutilization of cellular molecules [39, 40]. TFEB is a key regulator of the lysosomal and autophagy pathway [15, 41]. It has been demonstrated that activation of autophagy and lysosomal activity during serum starvation is concomitant with the fragmentation of mitochondria [28, 29]. In fact, quiescent fibroblasts have shorter and fragmented mitochondria when compared to proliferating cells, which have elongated mitochondria and increased oxidative phosphorylation [28]. Addition of serum to starved HCT116 cells induces mitochondria fusion and tubulation as soon as 3hrs after the reintroduction [28]. In this study, we found that NPC1 mutant fibroblasts already had a significant fragmentation of mitochondria in the presence of serum when compared to the healthy cells. When HT and NPC1 mutant fibroblasts were cultured for 24hrs with serum starved media, HT cells showed a significant fragmentation of mitochondria, while NPC1 mutant fibroblasts did not show changes statistically significant. However, 3hrs post reintroduction of serum after 24hrs of serum starvation was enough to see a small but significant increase in mitochondria elongation in both HT and NPC1 mutant fibroblasts. For the NPC1 mutant fibroblasts, this finding is significant, because it is consistent with evidence that shows that turnover of dysfunctional mitochondria is induced by serum starvation [29, 42]. TFEB nuclear translocation was also confirmed in the serum starved fibroblasts. These results encouraged us to examine if the potential activation of lysosomal function and autophagy by trehalose [22, 32, 34, 35] could have an impact on mitochondria morphology and length. We not only found mitochondria changes after the combination of serum starvation with the addition of trehalose, but also mitochondria tubulation and elongation occurred in these NPC1 mutant fibroblasts in the presence of medium (CM). Similarly, in *Chip* mutant fibroblasts from ataxia patients, trehalose treatment restored mitochondria morphology and activated autophagy among other effects [33]. Nuclear TFEB was also found in trehalose treated HT and NPC1 fibroblasts. However, trehalose did not have the same effect on HT fibroblasts, suggesting that as previously reported, a stronger mitophagy induction is observed in cells with damaged mitochondria [29, 42], promoting mitochondria turnover and presumably enhancing mitochondria health.

Another intriguing finding was the significant reduction of lysosomal accumulation of Filipin^+^ cholesterol in NPC1 mutant fibroblasts after trehalose treatment. Previous studies in NPC1 mutant fibroblasts have shown that induction of autophagy by rapamycin, an inhibitor of the mTORC1 pathway, rescues autophagy defects and protects these cells against induced cytotoxicity [17]. However, although mTORC1 inhibitors like rapamycin and Torin1 significantly rescue autophagy and mitochondria defects in *in vitro* cell models, they are unable to reduce lysosomal cholesterol accumulation in NPC1 mutant fibroblasts [13, 17]. Contrary to these mTORC1 inhibitors, trehalose treatment activates autophagy through an mTORC1 independent activation of TFEB, more specifically through the inhibition of the AKT pathway [21]. Inhibition of AKT by other pharmacological drugs also promotes the clearance of aggregated material in a variety of models of lysosomal storage diseases [21], suggesting that trehalose is not only capable of activating TFEB and mitochondria elongation, but also promotes the clearance of lysosomal aggregated material. It is thought that the mechanism by which trehalose activates TFEB is by increasing the lysosomal osmotic pressure, which transiently induces membrane permeability of calcium that leads to the nuclear translocation of TFEB [32].

Given that trehalose had such an enhancing effect on mitochondria morphology and lysosomal cholesterol accumulation in NPC1 mutant fibroblasts, we decided to test the effects of trehalose in COSCs from *Npc1*^*nmf164*^ mice. Lysosomal and autophagy dysfunction leads to the degeneration of axons and dendrites and ultimately neuronal cell death of adult neurons [18, 19, 41]. Treatments that enhance lysosomal-autophagy activity, like TFEB activation, have proven to be beneficial in some neurodegenerative mouse models [21, 43]. However, because previous work in our laboratory has shown that NPC1 deficiency affects the postnatal development of PCs *in vivo* [11], we focused our study on developing PCs *ex vivo*.

First, we examined mitochondria length in PCs dendrites from *Npc1*^*nmf164*^ COSCs and found that mitochondria were significantly fragmented in the mutant PCs when compared to WT PCs. Next, we tested the trehalose treatment in WT and *Npc1*^*nmf164*^ COSCs and found that trehalose was detrimental for both genotypes of developmental PCs. Significant fragmentation of mitochondria was found in the dendrites of WT PCs treated with trehalose, and *Npc1*^*nmf164*^ PCs showed a remarkable degeneration of dendrites. These trehalose-treated cells also presented increased nuclear TFEB and higher density of LAMP1^+^ lysosomes, suggesting that trehalose treatment affected dendritic growth by increasing lysosomal activity. Similarly, PCs from nontreated *Npc1*^*nmf164*^ COSCs had increased density of LAMP1^+^ lysosomes, which mirrored our previous findings where increased nuclear translocation of TFEB was concomitant with increased LAMP1^+^ lysosomes and dendritic regression in PCs from postnatal *Npc1*^*nmf164*^ mice [11]. Still, trehalose treatment has been effective enhancing the cellular clearance of proteolipid aggregates and preventing neuropathology in mice with a genetic mutation for the lysosomal storage disorder Batten disease [21]. However, these mice have a more prolonged life span (over 8 months) than NPC1 mutant mice (3–4 months), and the trehalose treatment was provided by the end of postnatal development. In NPC1 mutant mice, PCs dendritic postnatal development is significantly affected [11]. The role of lysosomes and autophagy in neuronal development, specifically in the growth and patterning of dendrites, is not completely understood. Since trehalose treatment induced the fragmentation of mitochondria in the *ex-vivo* developing PCs, it was not surprising to find that dendritic growth in WT COSCs was significantly compromised. These results suggest that trehalose treatment in WT COCs is detrimental for developmental PCs, possibly by over activating the lysosomal-autophagy pathway through TFEB activation. In fact, it has been demonstrated that basal autophagy contributes to the growth and arborization of dendrites, while excessive activation of autophagy leads to the reduced growth and terminal branching of dendrites in *Drosophila* multidendritic (md) sensory neurons [44]. Previous work in our laboratory also showed that early overgrowth of dendrites and the followed regression and reduction of the dendritic tree in NPC1 mutant PCs during postnatal development were associated with overactivation of TFEB [11]. Therefore, our data suggest that NPC1 deficiency causes a metabolic imbalance between anabolic and catabolic pathways that lead to early dendritic growth deficits that precede neurodegeneration. Given that TFEB is already overactivated in NPC1 mutant PCs at postnatal and pre-symptomatic stages of the disease [11], trehalose treatment may not be effective rescuing the neuropathology associated with NPC at PCs post-developmental stages.

## Supporting information

S1 Fig Filament tracing of PCP-2-GFP Purkinje cells. Sample images showing the use of the Imaris Filament tool to trace the dendrites of PCP2-GFP PCs in 3D confocal images. Scale bar: (A) 20 μm.
(TIF)

S2 Fig TFEB nuclear translocation after serum starvation. A. HT and NPC1 mutant fibroblasts immunolabeled with TFEB and DAPI showing nuclear translocation of TFEB after 24hrs of SSM. Scale bar: (A) 30 μm.
(TIF)

S3 Fig No effects of trehalose treatment in mitochondria length in HT fibroblasts. A. HT fibroblasts immunolabeled with PDHE1and DAPI showing morphological changes in mitochondria after 24hrs of SSM supplemented with H_2_O as a vehicle, or 100mM trehalose plus 3hrs of CM. B. Quantitative analysis of the length of PDHE1^+^ mitochondria in HT fibroblasts after the different treatments described in (A). Data are presented as mean ± SEM, n = 30 mitochondria/treatment. Scale bar: (A) 5 μm.
(TIF)

S4 Fig TFEB nuclear translocation after trehalose treatment. A. HT and NPC1 mutant fibroblasts immunolabeled with TFEB and DAPI showing nuclear translocation of TFEB after 24hrs of SSM + 100mM trehalose. Scale bar: (A) 40 μm.
(TIF)

S5 Fig Trehalose treatment causes dendritic degeneration in NPC1 deficient COSCs. A. NPC1 deficient P10 + 4DIV COSCs immunolabeled with PDHE1 and DAPI showing dendrite degeneration and loss of mitochondria after 100mM trehalose treatment. Scale bar: (A) 30 μm.
(TIF)

S6 Fig TFEB nuclear translocation after trehalose treatment in WT COSCs. A. WT P10 + 4DIV COSCs immunolabeled with TFEB, CALB, and DAPI showing nuclear TFEB immunoreactivity in CALB^+^ PCs (PC DAPI^+^ nuclei are circled by dashed lines) after trehalose treatment. B. Quantitative analysis of CALB^+^ PCs with nuclear translocation of TFEB. Nuclei are stained with DAPI. Data are presented as mean ± SEM, n = 8 images from COSCs n = 3. **P < 0.01. Scale bar: (A) 5 μm.
(TIF)
