# Supplementary figures and images for: Trehalose enhances mitochondria deficits in human NPC1 mutant fibroblasts but disrupts mouse Purkinje cell dendritic growth ex vivo
Source: PLoS One. 2023 Nov 30;18(11):e0294312. doi: 10.1371/journal.pone.0294312 (PMC10688965; doi:10.1371/journal.pone.0294312)

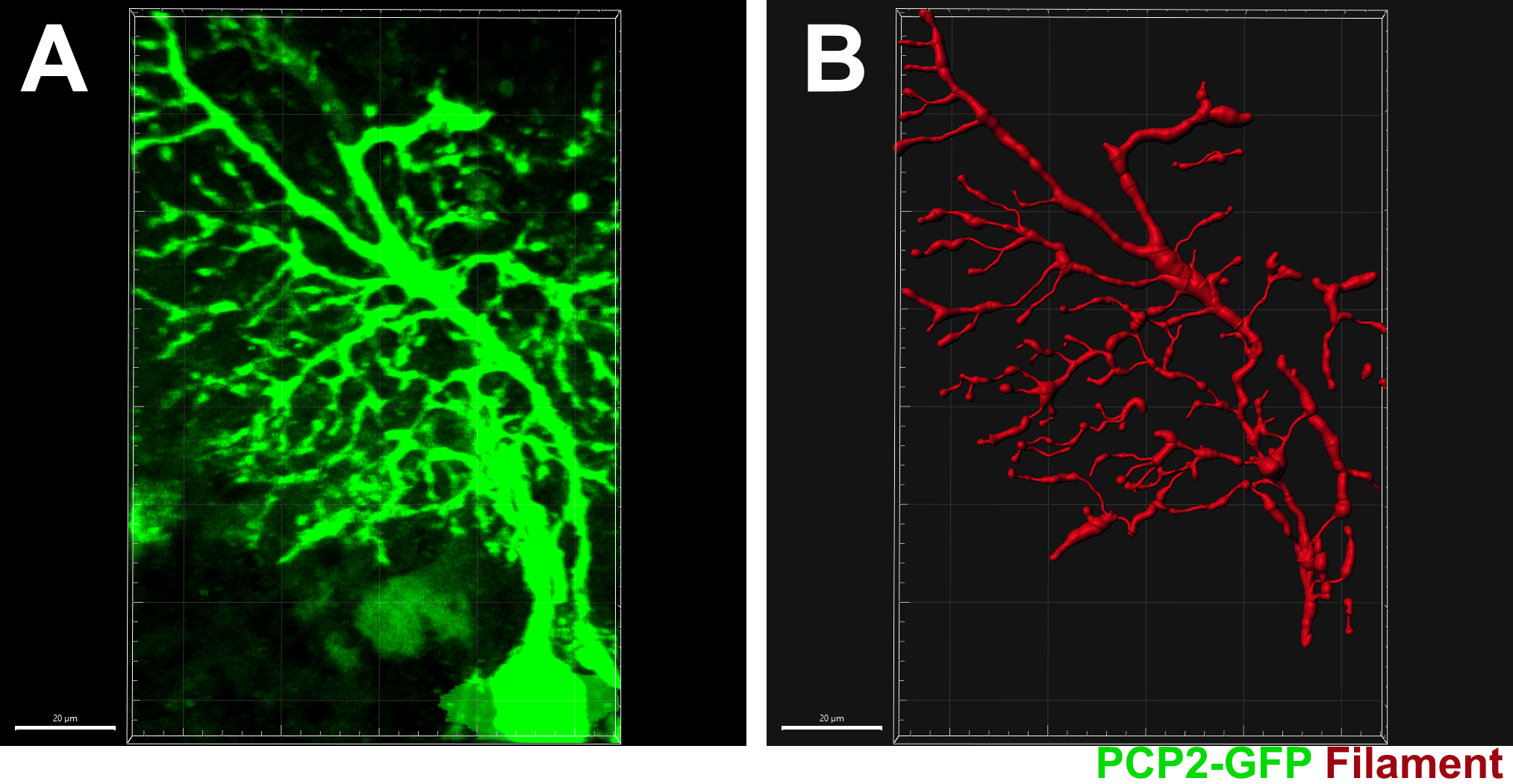

Supplement: S1 Fig — Sample images showing the use of the Imaris Filament tool to trace the dendrites of PCP2-GFP PCs in 3D confocal images. Scale bar: (A) 20 μm. (TIF) [file pone.0294312.s001.tif]

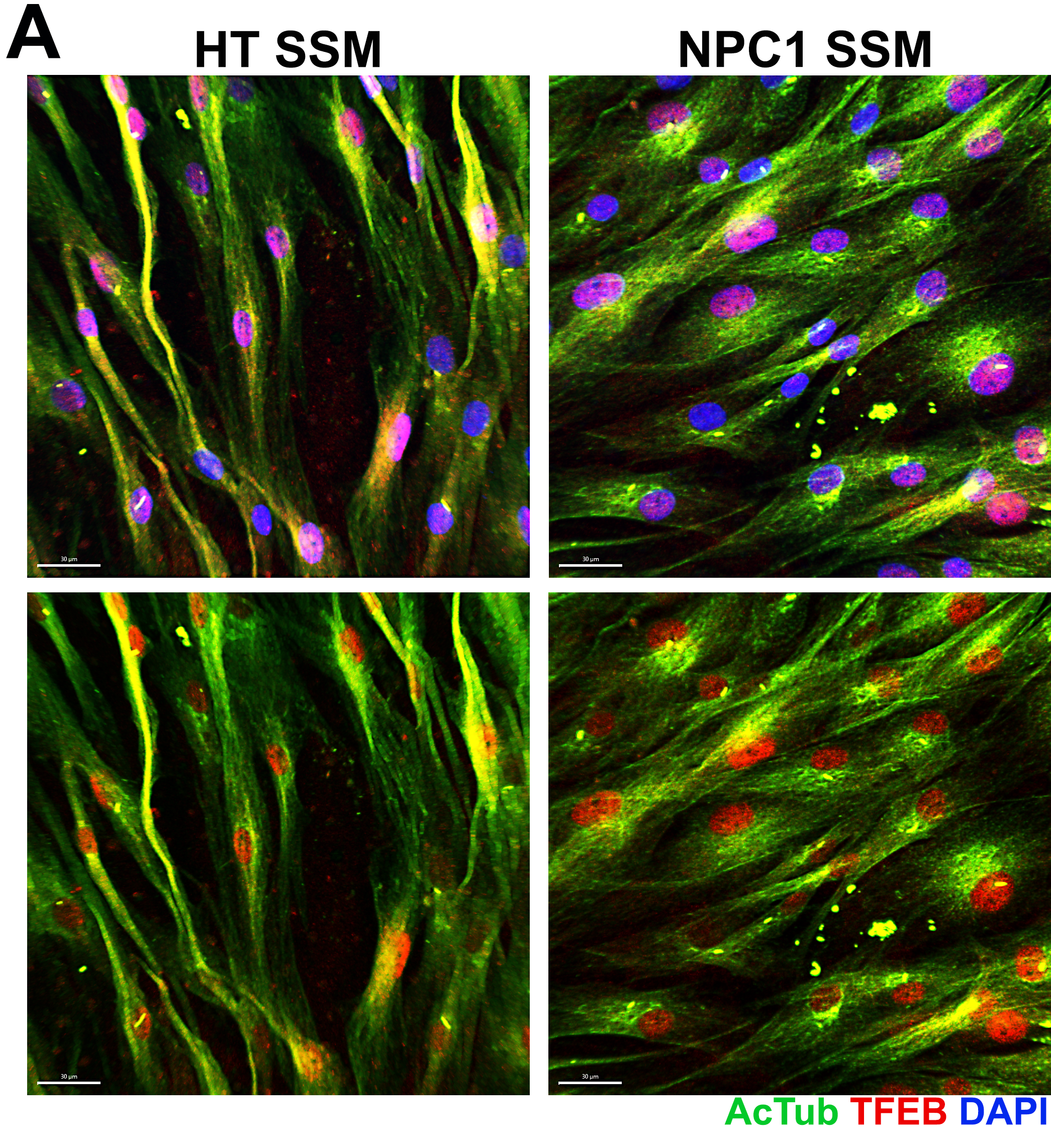

Supplement: S2 Fig — A. HT and NPC1 mutant fibroblasts immunolabeled with TFEB and DAPI showing nuclear translocation of TFEB after 24hrs of SSM. Scale bar: (A) 30 μm. (TIF) [file pone.0294312.s002.tif]

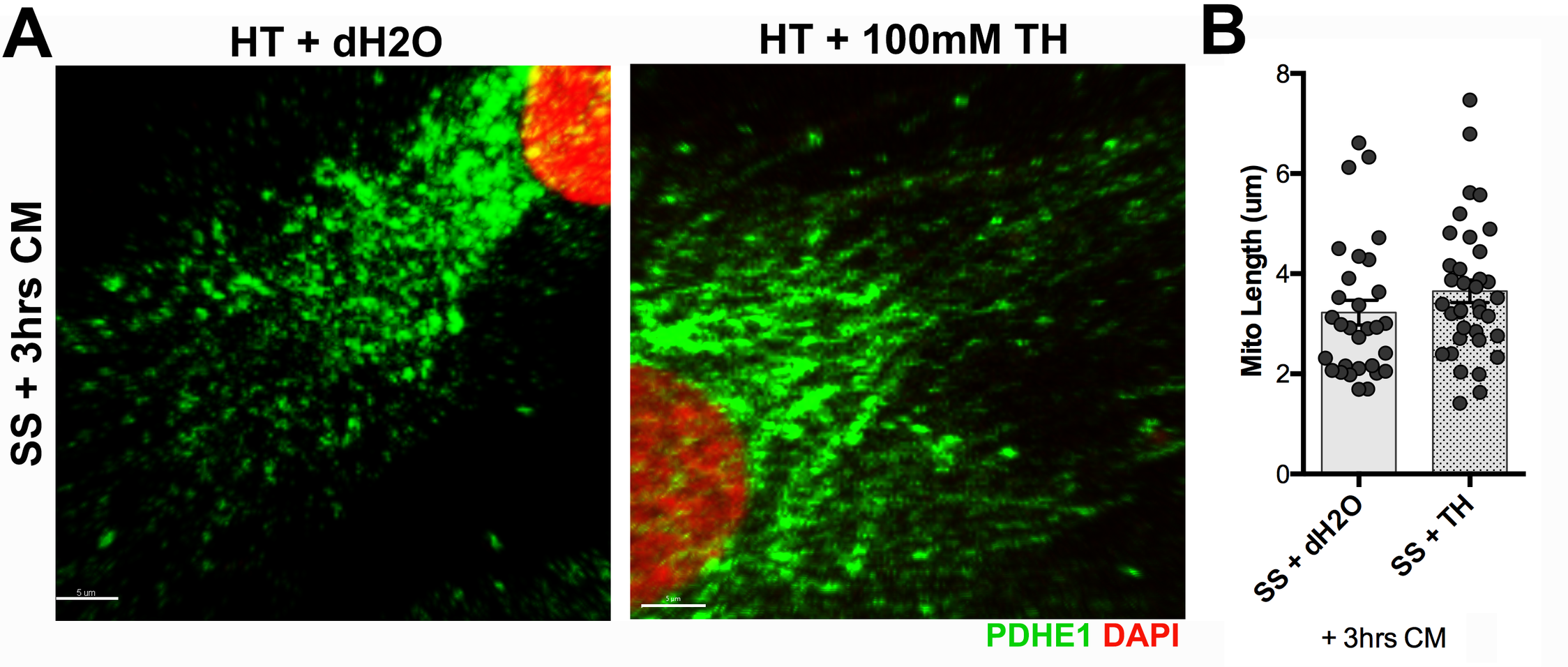

Supplement: S3 Fig — A. HT fibroblasts immunolabeled with PDHE1and DAPI showing morphological changes in mitochondria after 24hrs of SSM supplemented with H2O as a vehicle, or 100mM trehalose plus 3hrs of CM. B. Quantitative analysis of the length of PDHE1+ mitochondria in HT fibroblasts after the different treatments described in (A). Data are presented as mean ± SEM, n = 30 mitochondria/treatment. Scale bar: (A) 5 μm. (TIF) [file pone.0294312.s003.tif]

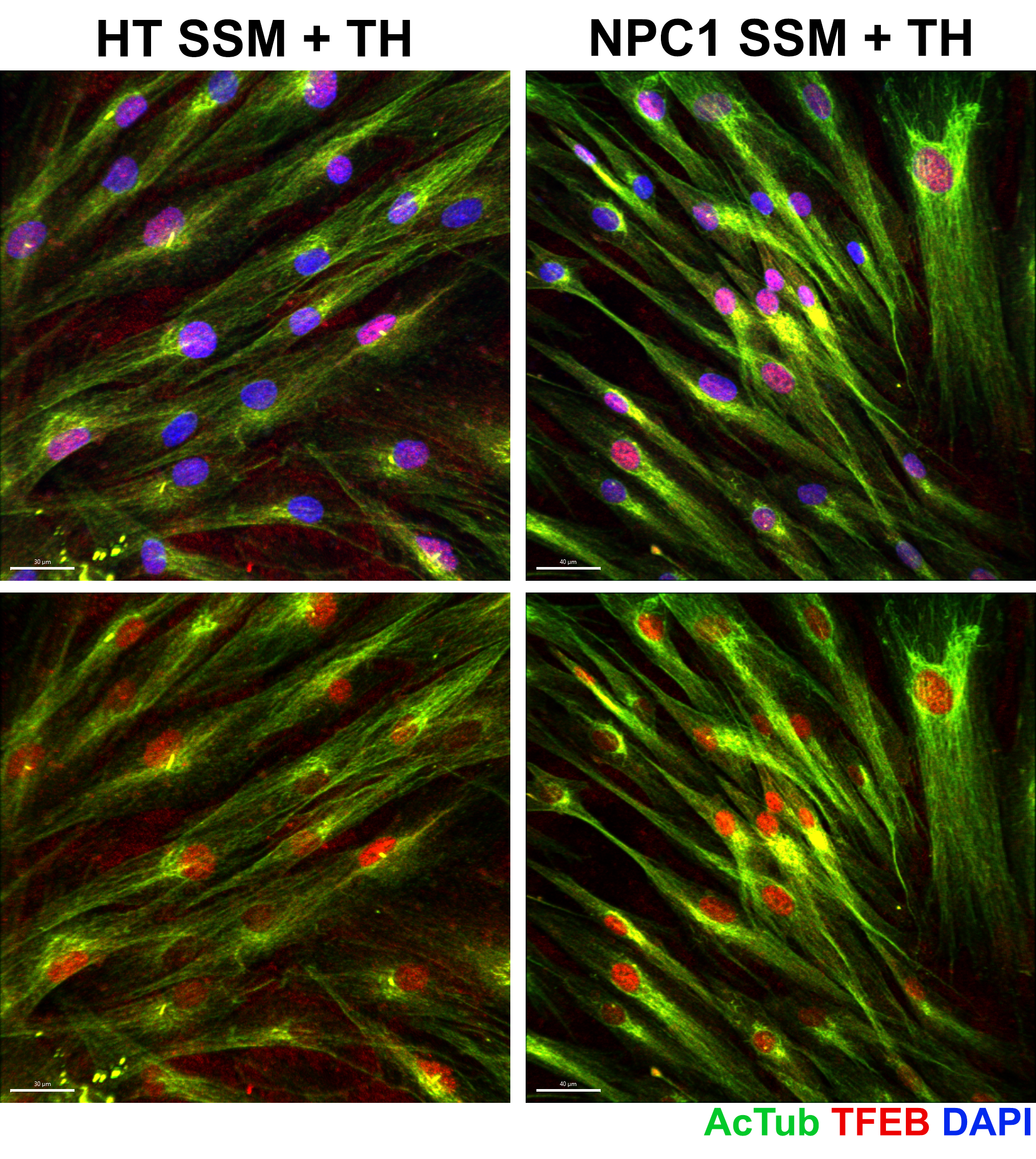

Supplement: S4 Fig — A. HT and NPC1 mutant fibroblasts immunolabeled with TFEB and DAPI showing nuclear translocation of TFEB after 24hrs of SSM + 100mM trehalose. Scale bar: (A) 40 μm. (TIF) [file pone.0294312.s004.tif]

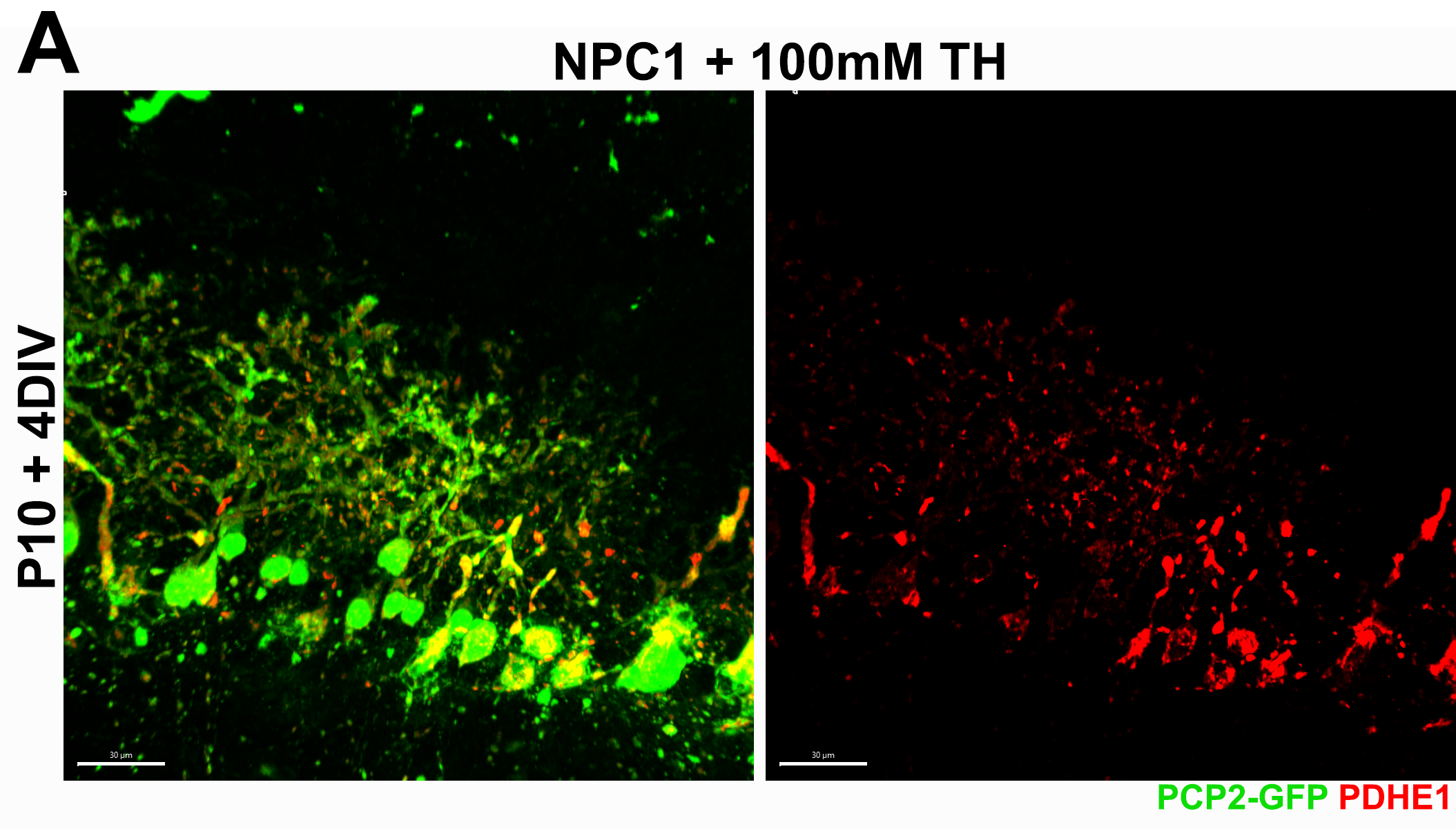

Supplement: S5 Fig — A. NPC1 deficient P10 + 4DIV COSCs immunolabeled with PDHE1 and DAPI showing dendrite degeneration and loss of mitochondria after 100mM trehalose treatment. Scale bar: (A) 30 μm. (TIF) [file pone.0294312.s005.tif]

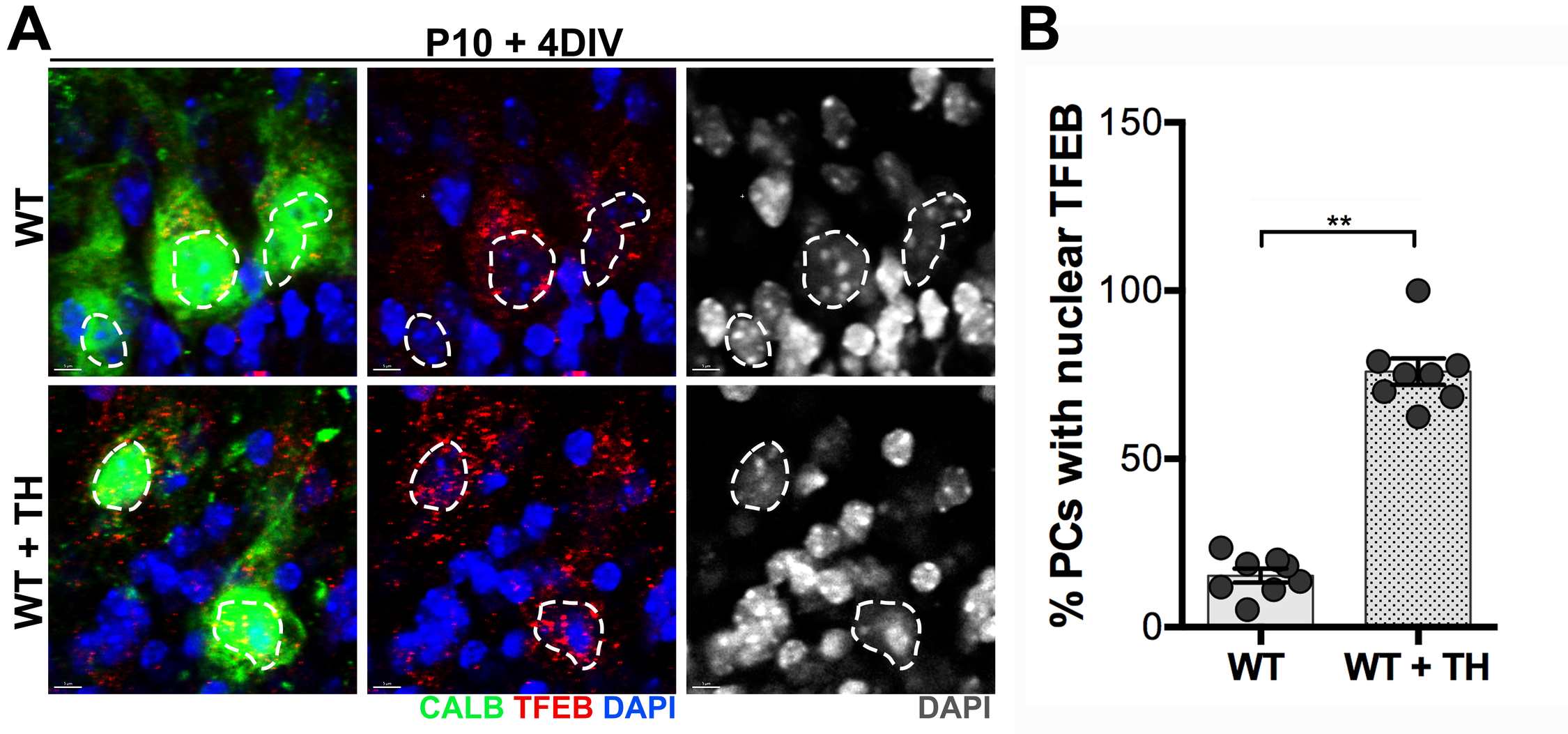

Supplement: S6 Fig — A. WT P10 + 4DIV COSCs immunolabeled with TFEB, CALB, and DAPI showing nuclear TFEB immunoreactivity in CALB+ PCs (PC DAPI+ nuclei are circled by dashed lines) after trehalose treatment. B. Quantitative analysis of CALB+ PCs with nuclear translocation of TFEB. Nuclei are stained with DAPI. Data are presented as mean ± SEM, n = 8 images from COSCs n = 3. **P < 0.01. Scale bar: (A) 5 μm. (TIF) [file pone.0294312.s006.tif]
